# Evaluation of vertical forces in the pads of Pitbulls with cranial cruciate ligament rupture

**DOI:** 10.1186/1746-6148-10-51

**Published:** 2014-03-01

**Authors:** Alexandre Navarro Alves Souza, Angelica Cecilia Tatarunas, Julia Maria Matera

**Affiliations:** 1 Department of Surgery, School of Veterinary Medicine and Animal Science, University of São Paulo (FMVZ/USP), São Paulo, SP, Brazil; 2 Department of Veterinary Medicine, School of Animal Science and Food Engineering, University of São Paulo (FZEA/USP), Pirassununga, SP, Brazil

**Keywords:** Vertical forces, Cranial cruciate ligament rupture, Dogs, Kinetic analysis, Pads

## Abstract

**Background:**

Cranial cruciate ligament rupture (CCLR) is one of the most important stifle injuries and a common cause of lameness in dogs. Our objective was to measure the vertical forces in the pads of Pitbulls with cranial cruciate ligament rupture (CCLR) using a pressure sensitive walkway. A pressure sensitive walkway was used to collect vertical force data from the pads of 10 Pitbulls affected with unilateral CCLR. Ten healthy Pitbulls were included in the study as controls. Velocity varied between 1.3 and 1.6 m/s and acceleration was kept below ± 0.1 m/s^2^. Differences between groups and between pads in the same limb within groups were investigated using ANOVA and the Tukey test. The paired Student t-test was employed to assess gait symmetry (p < 0.05).

**Results:**

Peak vertical forces (PVF) were lower in the affected limb, particularly in the metatarsal pad. Increased PVF values in the forelimb and the contralateral hind limb pads of affected dogs suggest a compensatory effect.

**Conclusions:**

A consistent pattern of vertical force distribution was observed in the pads of dogs with CCLR. These data are important for increased understanding of vertical force distribution in the limb of dogs with CCLR disease. Kinetic analysis using pressure sensitive walkways can be useful in follow-up assessment of surgically treated dogs regardless of the surgical technique employed.

## Background

Cranial cruciate ligament rupture is one of the most important stifle injuries and a common cause of lameness in dogs [1]. CCLR results in joint instability and leads to the development of degenerative joint disease over time [2-5]. Kinetic analysis is commonly employed for objective lameness evaluation in horses and dogs, among other species [6]. Peak vertical force (PVF) and vertical impulse (VI) are the most accurate parameters for lameness diagnosis [7] and can be measured using pressure sensitive walkways [8-10]. PVF and VI are significantly decreased in dogs with CCLR [6,11-14].

Vertical force redistribution studies in dogs with CCLR report a significant overload of the contralateral limb [9,10,15,16]. Similar to pedobarographic analysis in humans, the isolated analysis of specific areas of the limb during the stance phase of the stride can be performed in dogs using modern kinetic analysis equipment. Data obtained via these methods can be relevant when clinical decisions and patient follow-up are based on improved weight bearing (transfer of load through the paw to the rest of the limb) [17,18].

Studies on vertical forces in the pads of dogs [17,18] are scarce and unrelated to orthopedic disease. The aim of this study was to analyze vertical forces in the pads of dogs affected with CCLR. The description of PVF and VI in dogs with CCLR may contribute for a broader understanding of the changes that result in decreased weight bearing in these patients. It may also represent a more comprehensive method for patient follow-up and the critical evaluation of the surgical techniques currently employed to treat the condition.

## Methods

This research was approved by the Bioethics Committee of the Faculty of Veterinary Medicine and Animal Science of the University of São Paulo – FMVZ/USP. Ten healthy Pitbulls (control group) and 10 Pitbulls presenting with unilateral CCLR (CCLR group) were used in this study. Previous informed consent was given for the owners. All dogs were submitted to physical and radiographic examination, and the tibial thrust test prior to kinetic analysis. Dogs were aged between 2 and 6 years and weighed between 20 and 36 kg. Exclusion criteria were obesity, cachexia, pregnancy, estrous, history of previous orthopedic surgery, concurrent systemic or orthopedic disease, and medication of any kind over the preceding 4 weeks (minimum washout period of 4 weeks).

### Kinetic analysis

Kinetic analysis was performed on a 1.5 × 0.5-m pressure sensitive walkway^a^ equipped with a series of 3 plates instrumented with a total of 6864 sensors and connected to a dedicated computer.

Five valid trials were evaluated for each dog. Valid trials consisted of controlled velocity and acceleration in a straight line without sidestepping or deviation of the head. Out of a maximum of 20 consecutive passages recorded, 5 valid trials were selected for each dog. The first 4 passages were always excluded to avoid data collection before dogs were familiar with the setup. Only full stride cycles recorded in the middle of the platform were considered. The same operator (A.N.A.S.) was responsible for valid trail selection and analysis. To avoid potential interferences with kinetic analysis trials were always performed in the morning, before physical examination and before daily physical activities were resumed.

Before each session all sensors were calibrated according to a known standard weight. All trials were started 2 meters before the walkway, so that dogs had enough room to complete two full stride cycles before stepping on the platform. Walking velocity varied between 1.3 and 1.6 m/s and acceleration was kept below ± 0.1 m/s^2^. Velocity was given by the software as stride length divided by the duration of the stride cycle. Acceleration was controlled based on the difference between initial and final velocity divided by time. For increased strictness and to assure constant velocity, only stance phases with a variation of ± 0.01 seconds between consecutive foot strikes were considered for each leg. Dogs were walking fast during data collection in this study. Given gait analysis at the trot was not intended, only duty factors above 50% were considered. Duty factor ranged from 54.1 to 63.4% (mean, 58%).

Peak vertical force (PVF, Newtons) and vertical impulse (VI, N*s) were calculated from the vertical force curve generated automatically by the software^b^. For each foot strike evaluated, measurements of PVF and VI (expressed as percentage of body weight) were obtained from metacarpal/metatarsal pads and digital pads 2, 3, 4 and 5. These areas were manually outlined according to previously reported methods [18].

### Statistical analysis

Normal distribution of the data was investigated using the Kolmogorov-Smirnov test. Analysis of variance (ANOVA) and the Tukey test (post hoc) were used to compare the means and to assess the differences between groups and among pads in the same limb within each group. Gait symmetry between the right and left limbs in control dogs and between the healthy and the diseased limb in CCLR dogs was assessed using the paired Student’s t test. The level of significance was set at 5% (p <0.05). Sample power (difference between means based on standard deviation) greater than 80% confirmed the quality of the data.

## Results

The CCLR group consisted of 5 intact males and 5 intact females weighing 31.1 ± 3.9 kg and aged 4.2 ± 1.6 years. All dogs in this group had a history of lameness of at least 1 month duration (2.8 ± 1.5 months) but were not showing signs of acute lameness at the time of data collection. All dogs had been treated with non-steroidal anti-inflammatory drugs, but had completed the minimum washout period. The control group consisted of 4 intact males and 6 intact females weighing 28.8 ± 5 kg and aged 4.5 ± 1.2 years. Age and body weight did not differ between groups. Mean PVF and VI values expressed as percentage of body weight and respective standard deviations are summarized in Tables 1, 2 and 3.

**Table 1:** Peak vertical force (PVF) and vertical impulse (VI) for total of the limbs (mean ± SD)

**Control group**	**Forelimb**		**Hind limb**	
	**Right**	**Left**	**Right**	**Left**
PVF	54.6 ± 6.7^a^	55.2 ± 6.0^a^	34.2 ± 5.7^b^	33.4 ± 5.3^b^
VI	23.4 ± 2.9^a^	24.5 ± 3.3^a^	13.0 ± 1.6^b^	13.0 ± 1.4^b^
**CCLR group**	**Ipsilateral**	**Contralateral**	**Affected**	**Contralateral**
PVF	60.5 ± 6.1^c^	58.8 ± 6.7^c^	23.6 ± 7.4^d^	39.3 ± 6.0^e^
VI	25.7 ± 1.9^a^	27.0 ± 2.5^c^	7.7 ± 1.2^d^	16.8 ± 1.6^e^

PVF = peak vertical force; VI = vertical impulse. Groups with different letters in the same row are significantly different (p < 0.05). Mean values expressed as % of body weight.

**Table 2:** Peak vertical force (PVF) for the pads (mean ± SD)

**Forelimbs**	**Metacarpalpad**	**Digital pads**			
		**2**	**3**	**4**	**5**
Healthy	15.0 ± 3.5^aA^	5.6 ± 1.6^aB^	11.3 ± 1.7^aC^	12.6 ± 1.4^aD^	11.2 ± 1.9^aC^
Ipsilateral	22.3 ± 4.7^bA^	6.0 ± 1.4^aB^	12.6 ± 2.5^bC^	14.3 ± 2.2^bC^	15.1 ± 2.7^bC^
Contralateral	20.3 ± 5.3^cA^	5.9 ± 1.5^aB^	12.9 ± 2.5^bC^	14.3 ± 1.9^bC^	14.4 ± 2.6^cC^
**Hind limbs**	**Metatarsalpad**	**Digital pads**			
		2	3	4	5
Healthy	7.7 ± 2.2^aA^	3.1 ± 0.7^aB^	8.1 ± 1.3^aA^	9.1 ± 1.4^aC^	6.9 ± 1.3^aD^
Affected	2.5 ± 2.2^bA^	2.5 ± 1.2^bA^	7.7 ± 1.9^aB^	8.4 ± 2.9^aB^	4.1 ± 1.4^bC^
Contralateral	14.0 ± 3.2^cA^	5.0 ± 1.6^cB^	11.1 ± 2.0^bC^	10.9 ± 1.5^bC^	8.1 ± 2.0^cD^

Groups with different letters are significantly different (p < 0.05).

Capital letter- row/low case letter – column. Mean values expressed as % of body weight.

**Table 3:** Vertical impulse (VI)for the pads (mean ± SD)

**Forelimbs**	**Metacarpalpad**	**Digital pads**			
		**2**	**3**	**4**	**5**
Healthy	4.8 ± 0.7^aA^	1.8 ± 0.4^aB^	4.6 ± 0.7^aA^	5.5 ± 0.8^aA^	5.1 ± 0.8^aA^
Ipsilateral	7.1 ± 0.9^bA^	2.1 ± 0.3^aB^	4.6 ± 0.6^aC^	6.0 ± 0.6^aD^	6.0 ± 0.7^bD^
Contralateral	6.7 ± 1.2^bA^	2.3 ± 0.5^aB^	5.1 ± 0.6^aC^	6.2 ± 0.6^aD^	6.2 ± 0.8^bD^
**Hind limbs**	**Metatarsalpad**	**Digital pads**			
		2	3	4	5
Healthy	1.6 ± 0.3^aA^	0.7 ± 0.2^aB^	3.5 ± 0.5^aC^	4.4 ± 0.9^aD^	2.3 ± 0.4^aE^
Affected	0.5 ± 0.3^bA^	0.7 ± 0.2^aA^	2.5 ± 0.5^bB^	3.0 ± 0.5^bB^	1.2 ± 0.3^bC^
Contralateral	3.0 ± 0.4^cA^	1.6 ± 0.3^bB^	4.6 ± 0.5^cC^	4.8 ± 0.5^aC^	2.8 ± 0.5^cA^

Groups with different letters are significantly different (p < 0.05).

Capital letter- row/low case letter – column. Mean values expressed as % of body weight.

**Figure 1:**
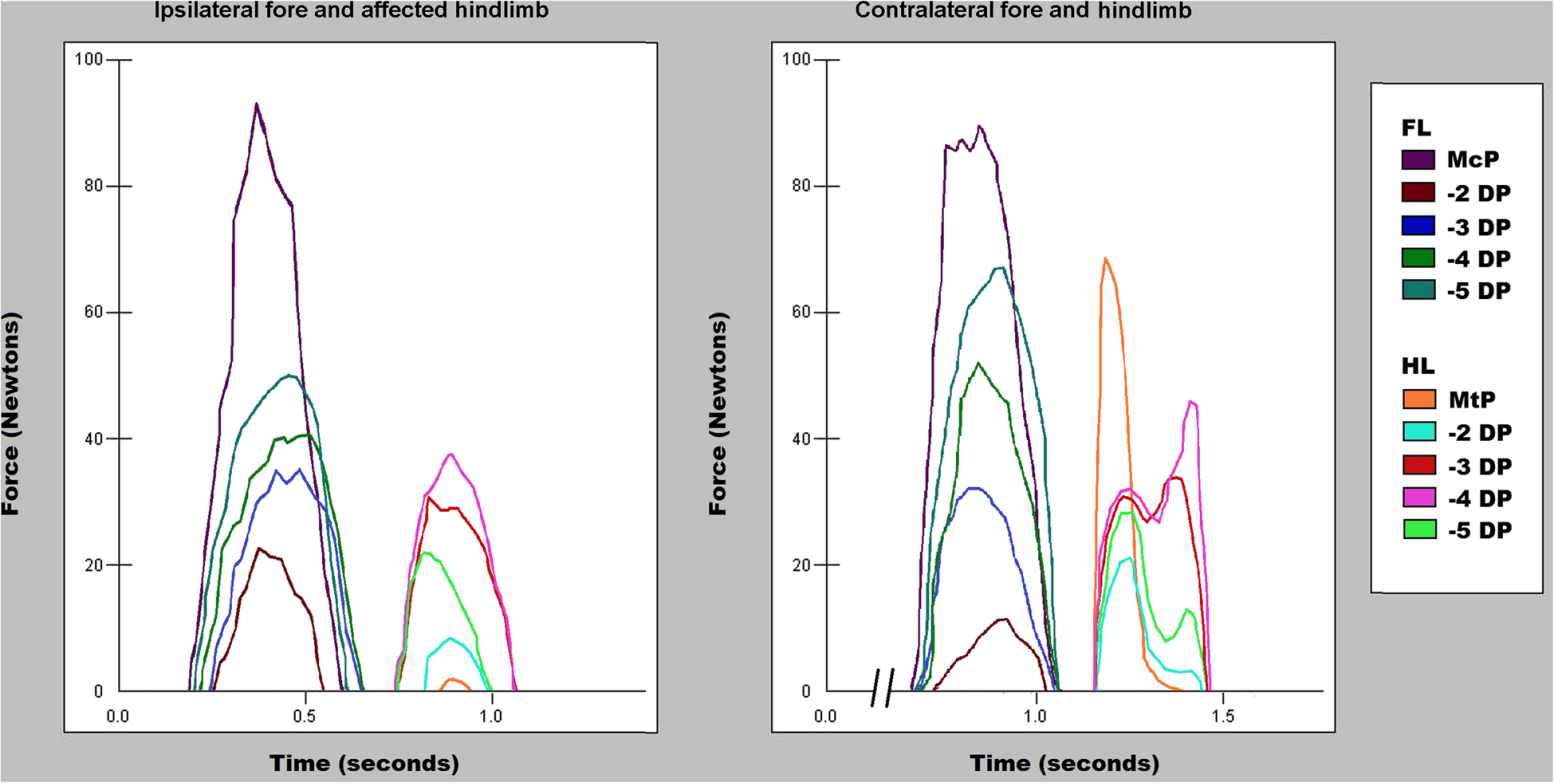
**Vertical force curve in the pads of a dog affected with CCLR.** Force curves reflect vertical force analysis of each footpad of a dog affected with CCLR during a valid passage. The same passage was sagitally separated to facilitate visualization and comparison between the affected and the contralateral hindlimb. The typical M-shaped waveform can be seen in the healthy, but not in the affected hindlimb. Peak vertical force is lower in the metatarsal pad of the affected hindlimb.

No gait asymmetries were observed in the control group (Table 1). However, important differences were observed in CCLR dogs. PVF and VI were lower in affected limb than in the contralateral limb and in the fore limbs when compared to control (Table 1), indicating that the affected limb, particularly the contralateral limb, is spared at the expense of the remaining limbs in cases of CCLR. Given no gait asymmetries were observed in the control group, the left and right front and hindlimbs were grouped together (i.e. healthy forelimb and hindlimb) and used as a reference for pad evaluation. In the CCLR group, the most prominent decrease in PVF was documented in the metatarsal pad of the affected limb. Mean PVF values in digital pads 3 and 4 were similar to mean values recorded in control dogs (Tables 2 and 3). Vertical force curves of healthy hindlimbs and hindlimbs with CCLR are shown in Figure 1.

## Discussion

Kinetic analysis is more sensitive than subjective evaluation for lameness diagnosis in dogs [19,20]. Although craniocaudal forces can also be measured using force plates, such forces are less reliable than PVF and VI for lameness diagnosis in dogs due to greater variability [7,20]. Vertical forces have 90% sensitivity and specificity for lameness detection and can be accurately documented using pressure sensitive walkways [8,9,21], as performed in this study.

Despite the wide popularity of kinetic analysis, studies on kinetic analysis in canine pads are scarce [17,18,22]. Changes in vertical forces lead to a decrease in PVF and VI in dogs with CCLR [10-16,23,24].

The few kinetic studies on load distribution in canine pads published to date report important contribution of the metatarsal pad for total weight bearing in German Shepherds [18], Labradors and Greyhounds [17]. In this study, the lower mean vertical forces documented in CCLR dogs reflected decreased weight bearing on the metatarsal pad in particular. While vertical forces in the affected limb corresponded to approximately 70% of the mean values documented in control dogs, vertical forces on the metatarsal pad were as low as 30% of controls.

The effect of breed on limb [25,26] and pad [17,18]kinetic analysis has been reported in dogs. Therefore dogs of the same breed were used in this study. All dogs had a history of lameness of at least 1 month duration. Lameness may be more severe in acute cases or shortly after surgery [27].

As previously reported, the classical M-shape of the vertical force curve reflects the specific dynamics involved in the stance phase of the stride, that begins with braking (footstrike) and ends with propulsion as the dogs lifts the limb off ground (toe off). The first vertical force peak corresponds to the maximum force generated during braking and is followed by a second peak representing the maximum force generated by propulsion. The valley between both peaks represents the movement of the limb from footstrike to toe off (mid-stance) [6,18]. The characteristic M-shaped pattern may be absent in faster gaits, particularly in the front limbs. A single force peak may then be visualized due to superimposition of the force peaks corresponding to footstrike and toe off respectively [18,22].

In this study, the M-shaped waveform typically seen during walking was observed in healthy hindlimbs, but not in hindlimbs affected with CCLR (Figure 1). Whenever the M-shaped waveform was observed the first vertical force peak was associated with the metacarpal or the metatarsal pad and the second with the digital pads, particularly the 3rd and 4th hindlimb pads.

Vertical forces are usually distributed among all pads during the stance phase of the stride in dogs. However, our results suggest that the aforementioned areas were possibly responsible for a higher percentage of weight bearing and for the braking and propulsion vertical force peaks. This may be related to CCLR given the braking phase of the stride is one of the most affected by the instability of the joint due to the cranial movement of the tibia [28], as simulated during physical examination using the tibial thrust test [29]. Vertical force magnitude in metatarsal pad may thus constitute an important parameter for post-surgical follow-up of CCLR cases. Vertical force measurements may also indirectly aid in the identification of residual joint instability with potential impact on weight bearing, as shown in *ex vivo* studies [30].

Weight bearing can also be measured based on craniocaudal force measurements although this method is less accurate due to lower force magnitudes and greater variability. Also, craniocaudal force measurements require the use of a force plate for evaluation of forces generated in three orthogonal planes during movement. Conversely, vertical forces may be measured in any pressure sensitive walkway at a lower cost.

A setup containing a series of instrumented plates capable of sampling a complete stride cycle during the same passage would reduce examination time and the degree of physical exertion required from subjects [9], while permitting consistent data collection [7,31]. Good quality portable craniocaudal force measurement systems and pressure sensitive walkways are currently available and yield reliable data despite differences in calibration and PVF readings [9].

## Conclusions

The results of this study suggest that evaluation of vertical forces in the pads using pressure sensitive walkways may be a promising method for evaluation of dogs with CCLR. The application of this diagnostic tool in other orthopedic diseases that are currently evaluated based on conventional kinetic analysis [32-35] may also contribute for increased understanding of the weight bearing changes observed in affected dogs.

The relevance of kinematic analysis of the tibiotarsal joint in dogs predisposed to CCLR has been reported [36] and important changes in weight bearing have been observed in the distal limb of affected dogs in this study. A comprehensive assessment of locomotion in these patients may be invaluable for critical evaluation of the surgical techniques currently employed to treat the condition.

## Endnotes

^a^7100 QL Virtual Sensor 3 Mat System, Tekscan Inc. South Boston, MA, USA.

^b^I-scan 5.231, Tekscan Inc., South Boston, MA,USA.

## Abbreviations

CCLR: Cranial cruciate ligament rupture; PVF: Peak vertical forces; VI: Vertical impulse.

## Competing interests

This study did not involve competing interests.

## Authors’ contribution

ANAS, ACT and JMM designed this study. ANAS and ACT examined all dogs involved. ANAS was responsible for kinetic data collection and analysis. ANAS and JMM prepared this manuscript. This manuscript was read and approved by all authors involved.

## References

[B1] Johnson JA Austin C Breur GJ Incidence of canine appendicular musculoskeletal disorders in 16 veterinary teaching hospitals from 1980 through 1989 Vet Comp Orthop Traumatol 1994 7 56 69

[B2] Comerford EJ Smith K Hayashi K Update on the aetiopathogenesis of canine cranial cruciate ligament disease Vet Comp Orthop Traumatol 2011 24 2 91 98 10.3415/VCOT-10-04-0055 21243176

[B3] Johnson KA Special issue on canine cruciate ligament disease Vet Comp Orthop Traumatol 2011 24 3 III IV 10.3415/VCOT-11-04-0063 21603740

[B4] Beraud R Moreau M Lussier B Effect of exercise on kinetic gait analysis of dogs afflicted by osteoarthritis Vet Comp Orthop Traumatol 2010 23 2 87 92 20151081 10.3415/VCOT-09-06-0068

[B5] Innes JF Bacon D Lynch C Pollard A Long-term outcome of surgery for dogs with cranial cruciate ligament deficiency Vet Rec 2000 147 12 325 328 10.1136/vr.147.12.325 11058021

[B6] Decamp CE Kinetic and kinematic gait analysis and the assessment of lameness in the dog Vet Clin North Am Small Anim Pract 1997 27 4 825 841 9243783 10.1016/s0195-5616(97)50082-9

[B7] Fanchon L Grandjean D Accuracy of asymmetry indices of ground reaction forces for diagnosis of hind limb lameness in dogs Am J Vet Res 2007 68 10 1089 1094 10.2460/ajvr.68.10.1089 17916016

[B8] Gibert S Lequang T Maitre P Cachon T Carozzo C Fau D Genevois J Viguier E Sensitivity and specificity to determine lameness in dogs with a pressure walkway system [Abstract] Proceedings of the 39th Annual Conference of the Veterinary Orthopedic Society; 2012 March 3–10 2012 25 Crested Butte CO, USA: Vet Comp Orthop Traumatol A21

[B9] Besancon MF Conzemius MG Derrick TR Ritter MJ Comparison of vertical forces in normal greyhounds between force platform and pressure walkway measurement systems Vet Comp Orthop Traumatol 2003 16 3 153 157

[B10] Oosterlinck M Bosmans T Gasthuys F Polis I Van Ryssen B Dewulf J Pille F Accuracy of pressure plate kinetic asymmetry indices and their correlation with visual gait assessment scores in lame and nonlame dogs Am J Vet Res 2011 72 6 820 825 10.2460/ajvr.72.6.820 21627529

[B11] Budsberg SC Verstrate MC Soutas-Little RW Flo GL Probst CW Force plate analysis before and after stabilization of canine stifles for cruciate injury Am J Vet Res 1988 49 9 1522 1524 3223659

[B12] Jevens DJ Decamp CE Hauptman J Braden TD Richter M Robinson R Use of force-plate analysis of gait to compare two surgical techniques for treatment of cranial cruciate ligament rupture in dogs Am J Vet Res 1996 57 3 389 393 8669774

[B13] Voss K Damur DM Guerrero T Haessig M Montavon PM Force plate gait analysis to assess limb function after tibial tuberosity advancement in dogs with cranial cruciate ligament disease Vet Comp Orthop Traumatol 2008 21 3 243 249 18536851

[B14] Böddeker J Drüen S Meyer-Lindenberg A Fher M Nolte I Wefstaed P Computer-assisted gait analysis of the dog: comparison of two surgical techniques for the ruptured cranial cruciate ligament Vet Comp Orthop Traumatol 2012 25 1 11 21 22105153 10.3415/VCOT-10-02-0025

[B15] Marsolais GS Dvorak G Conzemius MG Effects of postoperative rehabilitation on limb function after cranial cruciate ligament repair in dogs J Am Vet Med Assoc 2002 220 9 1325 1330 10.2460/javma.2002.220.1325 11991410

[B16] Ballagas AJ Montgomery RD Henderson RA Gillette R Pre and postoperative force plate analysis of dogs with experimentally transected cranial cruciate ligaments treated using tibial plateau leveling osteotomy Vet Surg 2004 33 2 187 190 10.1111/j.1532-950x.2004.04027.x 15027981

[B17] Besancon MF Conzemius MG Evans RB Ritter MJ Distribution of vertical forces in the pads of greyhounds and labrador retrievers during walking Am J Vet Res 2004 65 11 1479 1501 10.2460/ajvr.2004.65.1479 15566087

[B18] Souza AN Pinto AC Marvulle V Matera JM Evaluation of vertical forces in the pads of German shepherddogs Vet Comp Orthop Traumatol 2013 26 1 6 11 23111688 10.3415/VCOT-11-07-0100

[B19] Waxman AS Robinson DA Evans RB Hulse DA Innes JF Conzemius MG Relationship between objective and subjective assessment of limb function in normal dogs with an experimentally induced lameness Vet Surg 2008 37 3 241 246 10.1111/j.1532-950X.2008.00372.x 18394070

[B20] Quinn MM Keuler NS Lu Y Faria ML Muir P Markel MD Evaluation of agreement between numerical rating scales, visual analogue scoring scales, and force plate gait analysis in dogs Vet Surg 2007 36 4 360 367 10.1111/j.1532-950X.2007.00276.x 17547599

[B21] Lascelles BD Roe SC Smith E Reynolds L Markham J Marcellin-Little D Bergh MS Budsberg SC Evaluation of a pressure walkway system for measurement of vertical limb forces in clinically normal dogs Am J Vet Res 2006 67 2 277 282 10.2460/ajvr.67.2.277 16454633

[B22] Marghitu DB Swaim SF Rumph PF Cojocaru D Gillette RL Scardino MS Dynamics analysis of ground contact pressure of English pointer dogs Nonlinear Dynamics 2003 33 253 256 10.1023/A:1026096111497

[B23] Robinson DA Mason DR Evans R Conzemius MZ The effect of tibial plateau angle on ground reaction forces 4-17 months after tibial plateau leveling osteotomy in labrador retrievers Vet Surg 2006 35 3 294 299 10.1111/j.1532-950X.2006.00147.x 16635011

[B24] Dupuis J Harari J Papageorges M Galina AM Ratzlaff M Evaluation of fibular head transposition for repair of experimental cranial cruciate ligament injury in dogs Vet Surg 1994 23 1 1 12 10.1111/j.1532-950X.1994.tb00436.x 8140733

[B25] Voss K Wiestner T Galeandro L Hassig M Montavon PM Effect of dog breed and body conformation on vertical ground reaction forces, impulses, and stance times Vet Comp Orthop Traumatol 2011 24 2 106 112 10.3415/VCOT-10-06-0098 21243175

[B26] Molsa SH Hielm-Bjorkman AK Laitinen-Vapaavuori OM Force platform analysis in clinically healthy Rottweilers: comparison with Labrador Retrievers Vet Surg 2010 39 6 701 707 20345537 10.1111/j.1532-950X.2010.00651.x

[B27] Vaughan LC The history of canine cruciate ligament surgery from 1952–2005 Vet Comp Orthop Traumatol 2010 23 6 379 384 21155164

[B28] Ragetly CA Griffon DJ Mostafa AA Thomas JE Hsiao-Wecksler ET Inverse dynamics analysis of the pelvic limbs in labrador retrievers with and without cranial cruciate ligament disease Vet Surg 2010 39 4 513 522 10.1111/j.1532-950X.2010.00680.x 20345530

[B29] Harasen G Diagnosing rupture of the cranial cruciate ligament Can Vet J 2002 43 6 475 476 12058576 PMC339306

[B30] Hoffmann DE Kowaleski MP Johnson KA Evans RB Boudrieau RJ Ex vivo biomechanical evaluation of the canine cranial cruciate ligament-deficient stifle with varying angles of stifle joint flexion and axial loads after tibial tuberosity advancement Vet Surg 2011 40 3 311 320 10.1111/j.1532-950X.2011.00807.x 21361990

[B31] Nordquist B Fischer J Kim SY Stover SM Garcia-Nolen T Hayashi K Liu J Kapatkin AS Effects of trial repetition, limb side, intraday and inter-week variation on vertical and craniocaudal ground reaction forces in clinically normal labrador retrievers Vet Comp Orthop Traumatol 2011 24 6 435 444 10.3415/VCOT-11-01-0015 21938309

[B32] Gillette RL Angle TC Recent developments in canine locomotor analysis: a review Vet J 2008 178 2 165 176 10.1016/j.tvjl.2008.01.009 18406641

[B33] Madore E Huneault L Moreau M Dupuis J Comparison of trot kinetics between dogs with stifle or hip arthrosis Vet Comp Orthop Traumatol 2007 20 2 102 107 17546210 10.1160/vcot-06-06-0052

[B34] Burton NJ Dobney JA Owen MR Colborne GR Joint angle, moment and power compensations in dogs with fragmented medial coronoid process Vet Comp Orthop Traumatol 2008 21 2 110 118 18545712 10.3415/vcot-07-04-0038

[B35] Drüen S Böddeker J Meyer-Lindenberg A Fehr M Nolte I Wefstaedt P Computer-based gait analysis of dogs: evaluation of kinetic and kinematic parameters after cemented and cementless total hip replacement Vet Comp Orthop Traumatol 2012 25 5 375 384 10.3415/VCOT-10-02-0026 22828804

[B36] Ragetly CA Griffon DJ Klump LM Hsiao-Wecksler ET Pelvic limb kinetic and kinematic analysis in labrador retrievers predisposed or at a low risk for cranial cruciate ligament disease Vet Surg 2012 41 8 973 982 10.1111/j.1532-950X.2012.01042.x 23198924

